# Learning about learning: Mining human brain sub-network biomarkers from fMRI data

**DOI:** 10.1371/journal.pone.0184344

**Published:** 2017-10-10

**Authors:** Petko Bogdanov, Nazli Dereli, Xuan-Hong Dang, Danielle S. Bassett, Nicholas F. Wymbs, Scott T. Grafton, Ambuj K. Singh

**Affiliations:** 1 Department of Computer Science, University at Albany—SUNY, 1400 Washington Ave, Albany, NY 12222, United States of America; 2 Ticketmaster, Los Angeles, CA, United States of America; 3 Department of Computer Science, University of California Santa Barbara, Santa Barbara, CA 93106-5110, United States of America; 4 Complex Systems Group, Department of Bioengineering, University of Pennsylvania, Philadelphia, PA, 19104, United States of America; 5 Department of Electrical Engineering, University of Pennsylvania, Philadelphia, PA, 19104, United States of America; 6 Department of Physical Medicine and Rehabilitation, Johns Hopkins Medical Institutions, Baltimore, MD 21205, United States of America; 7 Department of Psychology and UCSB Brain Imaging Center, University of California Santa Barbara, Santa Barbara, CA, United States of America; University of Texas at Austin, UNITED STATES

## Abstract

Modeling the brain as a functional network can reveal the relationship between distributed neurophysiological processes and functional interactions between brain structures. Existing literature on functional brain networks focuses mainly on a battery of network properties in “resting state” employing, for example, modularity, clustering, or path length among regions. In contrast, we seek to uncover functionally connected subnetworks that predict or correlate with cohort differences and are conserved within the subjects within a cohort. We focus on differences in both the rate of learning as well as overall performance in a sensorimotor task across subjects and develop a principled approach for the discovery of discriminative subgraphs of functional connectivity based on imaging acquired during practice. We discover two statistically significant subgraph regions: one involving multiple regions in the visual cortex and another involving the parietal operculum and planum temporale. High functional coherence in the former characterizes sessions in which subjects take longer to perform the task, while high coherence in the latter is associated with high learning rate (performance improvement across trials). Our proposed methodology is general, in that it can be applied to other cognitive tasks, to study learning or to differentiate between healthy patients and patients with neurological disorders, by revealing the salient interactions among brain regions associated with the observed global state. The discovery of such significant discriminative subgraphs promises a better data-driven understanding of the dynamic brain processes associated with high-level cognitive functions.

## Introduction

Network-based modeling and characterization of brain architectures has provided both a framework for integrating imaging data as well as for understanding the function and dynamics of the brain. Brain networks are traditionally constructed either from structural or functional imaging data. Functional brain networks represent the associations between regions estimated by statistical similarities in regional time series, as measured by correlation or coherence [1–4]. In the case of fMRI data, regional gray matter activity is measured by the *blood oxygenation level dependent (BOLD)* signal.

Brain networks are commonly studied using techniques drawn from graph theory and machine learning [5]. These techniques provide fundamental and generalizable mathematical representations of complex neuroimaging data: nodes represent brain regions and edges represent structural or functional connectivity. This simplified graphical representation enables the principled examination of patterns of brain connectivity across cognitive and disease states [6]. While the majority of network-based studies have focused on the brain’s resting state [7], more recent efforts have turned to understanding brain connectivity elicited by task demands, including visual processing [1, 2] and learning [3]. Global network analysis of both functional and structural connectivity has demonstrated that brain networks have characteristic topological properties, including dense modular structures and efficient long-distance paths [8, 9].

Traditional network analysis tools are not necessarily sensitive to small perturbations in functional or structural connectivity as they rely on network-wide statistics [10]. Recent efforts have focused on developing new algorithms to identify specific subgraphs that are discriminative between brain states (cognitive or disease) and therefore critical for an understanding of local neurophysiological processes. Zalesky and colleagues describe a set of methods to identify groups of edges that are significantly different between two groups of networks [11, 12]. Kim et al. recently proposed powerful statistical tests for identifying functional edges that differ between groups [13, 14]. Motifs, defined patterns of local connectivity that occur frequently across sessions and subjects, are groups of edges with particular topological properties that may play specific functional roles [15, 16]. Hyperedges, considered by Bassett and colleagues, were defined as groups of edges that vary significantly in weight over time [17], for example during adaptive functions like learning or during higher-order cognitive processes like memory and attention [18]. In general, all of these tools seek to associate local network features (or subgraphs) with cognitive function, offering fundamental understanding and the opportunity to inform therapeutic interventions.

Here, we take an approach that is complementary to previous approaches focused on individual brain regions or single connections [12, 13, 15, 19, 20]. Our focus is on the network architecture of the brain, and how that architecture relates to behavior. Specifically, in this study, we develop and apply a novel analysis framework for identifying subgraphs that discriminate between individuals with differing behavioral variables. Our approach is: (i) data-driven in that it does not make assumptions about network sparsity, edge independence, etc.; (ii) enforces subnetwork connectivity in both the discovery and statistical scoring of discriminative subnetworks; and (iii) draws on novel machine learning methods for network state classification. Particularly, we extend recent techniques from labeled network mining [21], whose goal is to classify network instances based on individual node/edge states. In our setting, the goal is uncover a network-specific type of *biomarker*—a connected subgraph of functional edges—whose coherence predicts whether individuals are learning a motor sequencing task at a *high* or *low* rate. We learn a low-dimensional subspace of connected brain regions that discriminates among the categories representing high and low learning rates. To ensure generalization in the presence of few training instances, we build multiple models by performing k-fold validation and repeating the fold partitioning multiple times. We then mine conserved discriminative subnetworks across runs using frequent subgraph mining [22] and employ randomized statistical tests to establish the significance (in terms of q-value [23]) of the discovered subgraphs while implementing a *false discovery rate* correction for multiple comparisons.

We employ our framework to uncover the subgraphs that maximize the discriminative potential in explaining the differences in the rate of motor learning between individuals. The data for our analysis comes from a motor learning task in which subjects’ neural activity was measured using fMRI in repeated sessions as they learned a set of 12-note finger sequences [3]. Subjects learned three different sequences, each of which was presented as a string of spatial notes on a 4-line tablature. Each line corresponded to one of four fingers. The performance measure was movement time, which is the time required to perform a given 12-note sequence. We assign individual sessions to two categories—*high* and *low* rate learner sessions based on the measured slopes of learning rate. Intuitively, if a subject’s time in a given session decreases significantly, we classify the subject in this session as high-rate learner. The fMRI data was aligned to the Harvard-Oxford Brain Atlas (part of the FSL tool [24]) involving 112 cortical and subcortical regions. A functional edge strength linking two cortical areas was estimated as the wavelet-based coherence [4] of the corresponding regional time series.

Our work is the first to propose a general methodology for identifying network-based biomarkers for learning rate based on connected subgraph mining. Amongst tens of thousands of possible edges between 112 brain regions, we find connected functional subgraphs comprised of 1-5 edges that predict whether a subject is a high or low rate learner. These learning biomarkers agree with observations from previous studies [25–27] and further suggest new brain region relations which are essential to learning. Our proposed methodology is general in that it can be applied for studying the differences in other kinds of cognitive states or functional connectivity differences between disease and controls.

## Materials and methods

All human participants provided written informed consent after the study was approved by the University of California Santa Barbara Institutional Review Board. Our goal is to detect a set of functional edges interconnecting cortical regions whose coherence can predict differences among subjects in fMRI cognitive or disease-related studies. In the context of our data, the goal is to predict individual learning rates. We expect that learning-related changes in functional connectivity will be located in coordinated neural circuits involving co-activated regions forming a connected component [28–30], and we therefore restrict our attention to predictive edges that form a connected subgraph. As the rate of learning increases, some functional edges within a subgraph of interest will fall into a low coherence state (i.e., coherence between their adjacent regions’ activation will approach 0), while others will move into a high coherence state. We seek to understand these dynamics and extract structures (in the form of functional subgraphs) that predict the global behavioral state of the individual: high/low rates of learning that are statistically significant.

*Definition:* A subnetwork *biomarker* is a statistically significant connected subgraph of functional edges whose coherence states can collectively *differentiate* between cognitive scores (e.g., learning rate) of subjects.

**Fig 1:**
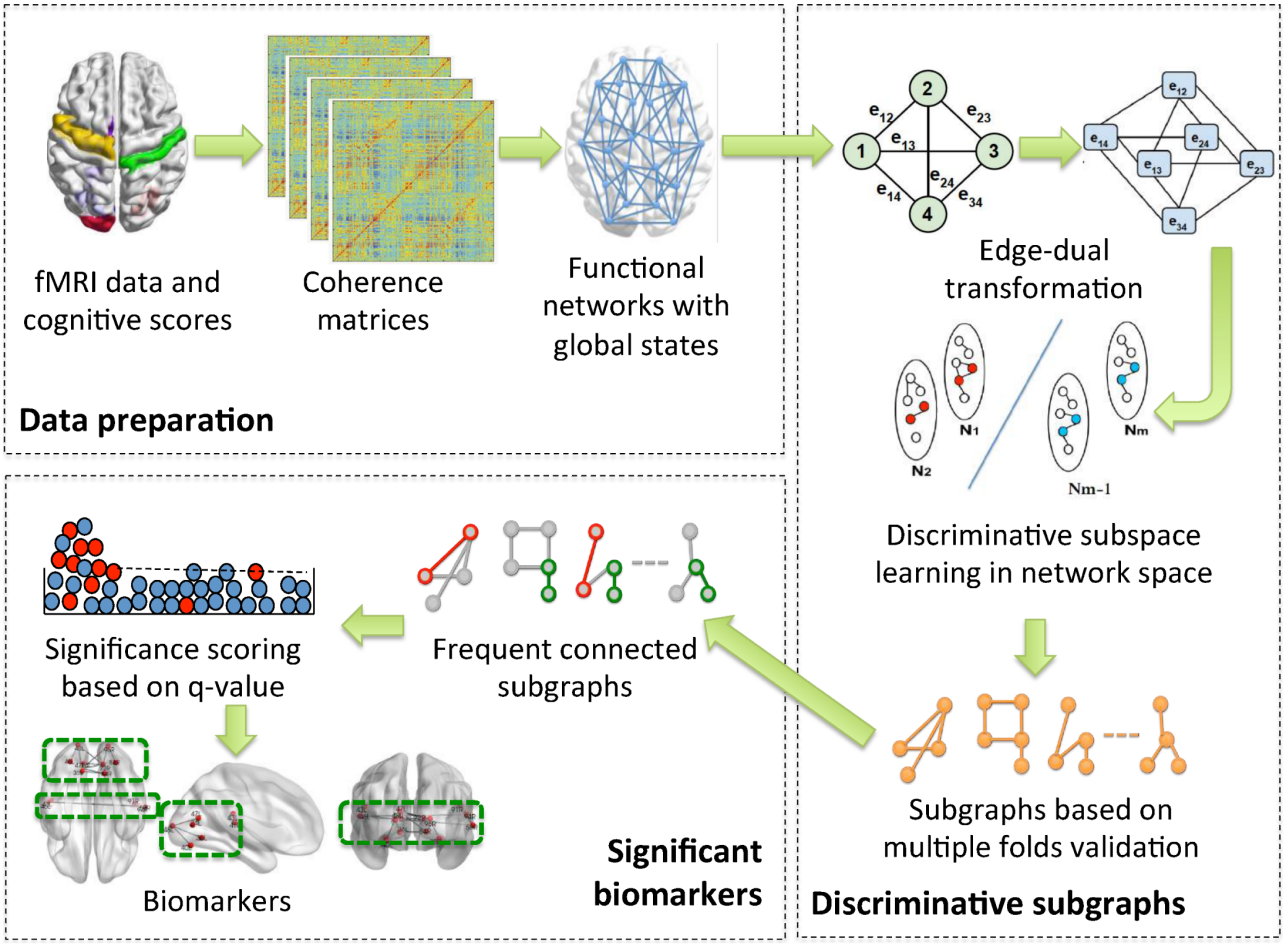
An overview of the method. We start with experimental fMRI and learning rate measurements from individual sessions. We compute pairwise coherence among all regions and create session-specific functional networks with a global label based on the learning rate. We transform the coherence networks into edge-dual graphs and perform a discriminative subspace learning with multiple-split k-fold validation to produce a set of discriminative connected subgraphs. Next, we mine conserved connected subgraphs identified by subspace learning and compute the significance of their individual accuracy while implementing a false discovery rate correction for multiple comparisons to obtain our final biomarkers.

The potential of a biomarker to differentiate between functional networks corresponding to low or high learning rate subject sessions is called *discriminative power* (and the biomarker is called *discriminative*). An overview of our approach is presented in Fig 1. We employ a discriminative biomarker mining approach to analyze functional networks constructed from fMRI scans of 18 subjects performing a motor learning task over 3 learning sessions that occurred on 3 different days. The sessions are divided into low and high learning rate sessions based on the average reduction in movement time to complete the motor task. The goal of our analysis is to identify biomarkers (subgraphs) that are *discriminative* of the session type (high *vs.* low rate learners), while at the same time statistically significant. We describe the steps in our approach in more detail in what follows.

### Data acquisition and preparation

The data for our analysis was collected during a motor learning experiment in which subjects’ neural activity was measured using fMRI [3, 31]. The data was originally used to analyze the brain’s functional flexibility during learning. Here, we follow the same protocol for data preparation, but focus on subgraph biomarkers associated with learning. Next, we provide a description of the original experimental procedure, apparatus, imaging protocol and data processing.

**Experimental procedure.** While in supine position, subjects performed a cued sequence production (CSP) task using the four fingers of their left hand, thumb excluded. To maximize comfort and to provide an angled surface to position the response box, subjects were positioned inside the scanner with foam padding under their knees. Padding was also placed under the left arm to provide extra support when responding during the task. Subjects performed the CSP task by responding to visually cued sequences on the response box using their left hand. The sequences were presented in static form, as a series of 12 music notes on a 4-line music staff. Subjects were instructed to type the sequences, reading from left to right, so that the top line of the staff mapped to the leftmost finger and the bottom line mapped the rightmost finger. Each 12-element sequence contained 3 notes per line. Each trial began with a fixation ‘+’, displayed for 2 seconds. The complete 12-element sequence was presented immediately following the offset of the fixation ‘+’, and participants were instructed to initiate responding quickly and accurately. The sequence remained on screen for the responding duration, or a time limit of 8 seconds, whichever came first. After completion of a correct sequence, the notes were replaced with a fixation signal until the trial duration was reached. With any incorrect press, a verbal cue “INCORRECT” appeared and the participant waited for the next trial.

Subjects trained on 16 different sequences, with training divided into 3 levels of intensity. Of these, 3 sequences were trained frequently (189 trials/sequence), with training distributed for these “frequent” sequences evenly across the training sessions. In addition, a second set of three sequences were presented moderately for 30 trials, and a third set of ten additional sequences, were presented rarely between 4 and 8 trials during training.

Frequent sequences were practiced in blocks of 10 trials, with 9 out of 10 trials in a block belonging to the same frequent sequence, and the other trial belonging to one of the ten rarely trained sequences. Trials were presented using an event-related structure, with sequence trials separated using an interstimulus interval that ranged between 0 and 20 seconds, along with additional time remaining following the completion of the previous trial. To provide some motivation for good performance, after each block of trials, subjects received feedback that detailed the number of correct trials and the average movement time (defined as the time to complete a sequence) needed to complete a correct sequence in that block. Scan epochs lasted 40 trials (4 blocks, 345 scan TRs), and each training session contained 6 scan epochs (2070 scan TRs).

**Apparatus.** Task presentation and online behavioral data acquisition was handled using a Dell Latitude D620 laptop computer running MATLAB 7.1 (Mathworks, Natick, MA) and the Cogent 2000 toolbox. Key-press responses and response times were collected using a custom response box with fiber optic signal transduction connected to a response card (DAQCard-6024e, National Instruments, Austin, TX).

**Imaging protocol.** Functional MR images were collected using a 3T Siemens Trio with a 12-channel phased-array head coil. For each scan epoch, a single-shot echo planar imaging sequence sensitive to BOLD contrast was used to acquire 33 slices per scan (repetition time [TR]/echo time[TE] 2000/30 ms, 3 mm axial slice, 0.5 mm gap, flip angle of 90°, field of view 192 mm, 64 × 64 inplane acquisition matrix). Prior to the initial functional scan epoch, a high-resolution T1-weighted scan (TR/echo time 15.0/4.2 ms, flip angle of 9°, 3D acquisition, 0.89 axial slice, field of view 256 mm, 256 × 256 inplane acquisition matrix) was collected.

Image preprocessing was performed using the FMRIB (Oxford Centre for Functional Magnetic Resonance Imaging of the Brain) included in the Software Library (FSL) [24]. Functional imaging time series realignment was performed using the fully automated program MCFLIRT (Motion Correction using FMRIB’s Linear Image Registration Tool) with realignment to the middle time series image. Images were high-pass filtered (50*s* cutoff), and spatially smoothed 8*mm* with a Gaussian kernel. No temporal smoothing was used. Further, signal intensity was normalized across all functional volumes in order to control for possible fluctuations in across sessions. Functional volumes were normalized to the Montreal Neurological Institute (MNI)-152 template with affine transformation (12 DOF) using FLIRT (FMRIB’s Linear Image Registration Tool). We parcellated the brain into 112 cortical and subcortical regions using the Harvard-Oxford structural atlas as supplied with FSL in standard space (MNI-152). For each individual participant and for each of the 112 regions, we calculated the regional activation level time series by finding the mean across all voxels in a region.

**Subjects.** The study involved 18 paid young adult participants without formal training in playing a musical instrument, with normal vision, and without neurological or psychiatric disorders. The number and order of sequence trials was identical for all participants. All participants completed three training sessions in a five-day period.

**Functional networks and learning rate labels.** Edges between nodes represented the pairwise coherence of the average fMRI time series for a pair of brain regions [32]. More specifically, we estimated a magnitude squared wavelet coherence, which identified areas in time-frequency space where two time series co-varied in the frequency band 0.06-0.12 Hz (we used fixed binning of this interval). This measure of functional connectivity was estimated using the minimum-variance distortionless response method [33], and provides a measure of nonlinear functional association between any two time series. In using the coherence, which has been demonstrated to be useful in the context of fMRI neuroimaging data [4], we were able to measure frequency-specific linear relationships between time series.

The coherence matrix of every session corresponds to a fully connected graph (a *clique*) involving all brain regions as nodes and coherence values associated with edges. Apart from the coherence values, each session is also characterized by movement time—the time to complete the sequence. Motor learning is well-characterized by an exponential drop-off in movement time; early learning shows a fast rate of drop-off, and is well-fit by one exponential curve, and later learning shows a slower rate of drop-off, and is well-fit by a second exponential curve [34, 35]. Here we study early learning, taking place over 3 days of moderate practice, and therefore examine a single exponential fit of the movement time versus trial bin. The magnitude of the exponential drop-off parameter indicates the gradient of the learning slope, where a sharper drop-off in movement time corresponds to individuals who are faster learners in the session, and a less-sharp drop-off in movement time corresponds to individuals who are slower learners respectively [30, 36].

We derive a learning rate class label (high and low) for each session-specific network based on the drop-off in learning slope. To estimate the threshold between the two classes, we cluster the drop-off values in two groups multiple times and adopt the consistent pivot between clusters over multiple runs as a threshold. Details on the threshold estimation are provided in the Supporting Information (S1 File). It is worth mentioning that multiple levels of learning rate can also be accommodated within our framework, requiring only minor modifications, however we restrict the analysis to two classes due to the limited number of instances. Further discussion of multiple-class analysis is available in the following section.

### Mining discriminative subgraphs

As a result of the data preparation we obtain a set of *global-state networks*: graphs with local coherence values on edges and a global network state indicating whether high or low learning rate was observed in the corresponding session. The setting is similar to that in a classification task in machine learning in that we have a set of instances (functional coherence networks) characterized by features (coherence values) and associated with labels (learning rates). The distinctive aspect in global network state classification is that there is an inherent structure imposed on the features: the shared network topology. The aim in global state network classification and feature selection is to find small connected subgraphs involving the most discriminative nodes, whose labels predict the global state of network instances. In our case, these subgraphs will correspond to interconnected brain regions, whose patterns of pairwise coherence discriminates between high and low learning rates.

We employ our recently proposed method for global state classification [21] to mine candidate substructures. Prior to describing our method, let us briefly discuss existing techniques [21, 37, 38] that deal with global network state classifications. They typically work with node labels as opposed to edge labels. Hence, in order to employ those techniques, we represent our data in this common framework by transforming the original functional network into its edge-dual graph. Original functional edges become vertices in the edge-dual graph. Two vertices have a link between them if their corresponding edges in the original network shared a common end-node (region in the brain). To avoid confusion, we will use edges and nodes when discussing the original functional graph among brain regions (112 nodes and 6216 edges); and vertices and links to refer to the elements of the edge-dual graph (6216 vertices and 344988 links). Similar consideration of original edges as vertices in a line graph has been also adopted by others in the network analysis literature [39, 40].

**Fig 2:**
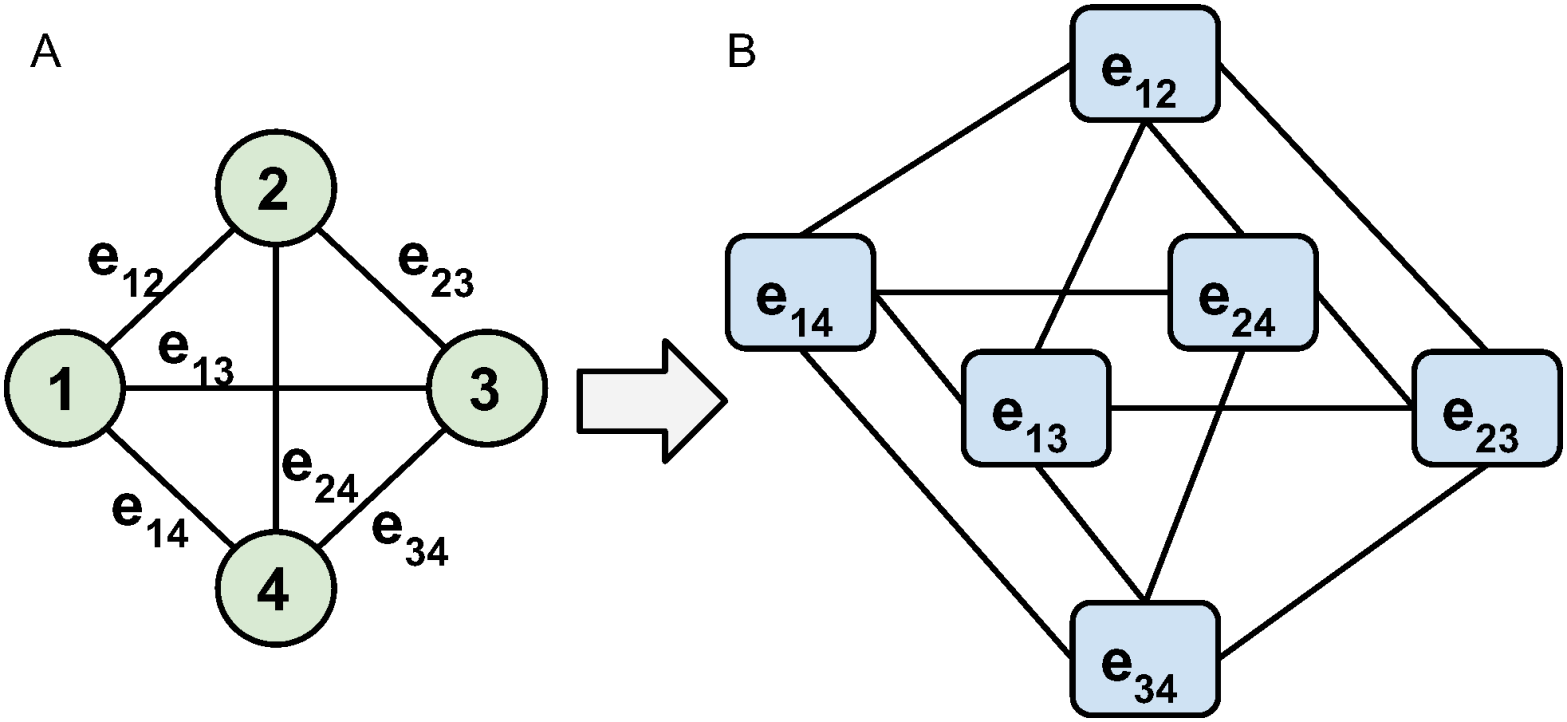
Edge dual graph. Transformation from edge-weighted graph (A) to a corresponding edge-dual graph (B). Edges between nodes become vertices and are connected by links if they share a node in the original graph.

The transformation is demonstrated in Fig 2 for a small example network of 4 nodes and 6 edges. If we start with a complete graph *G*(*N*, *E*) of |*N*| nodes and |*E*| = |*N*|(|*N*| − 1)/2 edges, the corresponding edge-dual graph *G*_*ed*_(*E*, *L*) will have |*E*| vertices and |*L*| = |*E*|(|*N*| − 2) links. In our example graph (Fig 2), we have 4 nodes and 6 edges and in the corresponding edge-dual graph we obtain 6 vertices and 12 links. In this transformation, originally adjacent edges become vertices in the dual graph and two vertices are connected by a link. Note that this transformation ensures that a subgraph of connected vertices in the dual graph corresponds to a connected subgraph of edges in the original functional network.

Discriminative subgraphs in a global network state setting can accurately differentiate between the global states. The space of potential discriminative subgraphs encompasses the set of all possible connected substructures of the functional brain network, which is exponential in the number of brain regions. Hence, the fair consideration of all subgraphs is computationally intractable even at the spatial resolution of 112 cortical and subcortical regions. Early methods in this area resort to sampling [37, 38]. While computationally efficient and ensuring the connectivity of discovered subgraphs, such approaches produce subgraphs that vary significantly across runs. To avoid such variation, we employ our recently introduced spectral learning method called *Sub-Network Learning (SNL)* [21] to produce candidate biomarkers. The key idea behind SNL is to project the original network instances to a low-dimensional subspace in which instances of different global states are well separated. Simultaneously, the method regularizes the learning process by enforcing locality in the edge-dual network topology within the projection. Consequently, dimensions of the learned low-dimensional subspace correspond to combinations of connected subnetworks with high global-state classification accuracy.

SNL constructs two meta-networks that capture the similarity relationships among network samples corresponding to individual sessions. It is important to note that vertices in these meta-networks correspond to edge-dual graphs, while a value associated with an edge represents the similarity between two edge-dual graphs. We denote with *G*^+^ the first meta-network, and with **A**^+^ its associated affinity matrix where an entry $A_{ij}^{+}$ captures the similarity between two session-specific graphs *i* and *j* that have *the same* global state value. Likewise, we use *G*^−^ to denote the second meta-network and **A**^−^ its associated affinity matrix where entry $A_{pq}^{-}$ captures the similarity between graphs *p* and *q* that have *different* global states.

In [21] we discuss several ways to compute the similarity among graph samples and for this study we adopt the cosine distance. Under this measure, all edge coherence values within a session are modeled as a vector and the pairwise cosine distances between session-specific vectors are used as weights in *G*^+^ and *G*^−^. Modeling more than two classes, e.g., different levels of learning rate, is possible within the framework where the semantics of *G*^+^ and *G*^−^ remain unchanged. For ordinal classes, i.e. learning rates with an order imposed on them, it might be desirable to apply an appropriate scaling for elements of *G*^−^ such that pairs of instances of “closer” labels incur smaller penalty. Prediction of continuous network labels (regression) would require a significant redesign of our learning framework, a direction we plan to explore in the future. It is important to note that higher number of classes or regression analysis would require much bigger training sets, hence we focus on two-class analysis for the sensorimotor experiment at hand.

Given the dissimilarity information encoded in *G*^+^ and *G*^−^, we learn a transformation function that maps graph samples from the original space to a *d*-dimensional latent subspace, of which graphs with the same global labels are mapped close to each other, while graphs with different global labels are rendered far apart. This objective ensures that graphs from different classes are well-discriminated. Learning this transformation function is further regularized by the graph topology, which promotes the inclusion of well-connected subgraphs that are related to the prediction of the global low and high learning rates.

SNL can learn an optimal subspace of arbitrary dimensionality *d* as long as *d* is smaller than the total number of vertices in the edge-dual graphs. However, similar to the approach of spectral clustering [41] or Fisher’s linear discriminant analysis [42], one often chooses *d* equal to one less than the number of expected clusters (or classes). Hence, to discriminate between low and high learning states, we project network samples onto a 1-dimensional subspace (i.e., *d* = 1). The dual-optimization function can be formulated as follows:

$$\begin{array}{cc} & \left\{ \begin{array}{c} \mathrm{minimize}\sum_{i=1}^{m}\sum_{j=1}^{m}{∥u^{T}v_{i}-u^{T}v_{j}∥}^{2}A_{ij}^{+}\\ \mathrm{maximize}\sum_{p=1}^{m}\sum_{q=1}^{m}{∥u^{T}v_{p}-u^{T}v_{q}∥}^{2}A_{pq}^{-} \end{array}\right.\\ & \;\;\;\;\;\;\;\;\;\;\;\;\;\;\;\;\;\;\;\;\;\;\;\;\;\;\;\text{subject}\;\text{to}\;\;u^{T}Cu\leq t\\ & \;\;\;\;\;\;\;\;\;\;\;\;\;\;\;\;\;\;\;\;\;\;\;\;\;\;\;\text{and}\;\;u^{T}VDV^{T}u=1, \end{array}$$ (1)

where *m* denotes the number of edge-dual graphs, and vector **v**_*i*_ stores vertex values (i.e., coherence) of the *i*-th graph sample. Session-specific vectors **v**_*i*_ comprise the columns of matrix **V**. Matrix **D** is diagonal with entries $D_{ii}=\sum_{j}A_{ij}^{+}$. Additionally, **C** is the combinatorial Laplacian matrix encoding the topology of the edge-dual graphs, and *t* is a parameter that captures the impact of this topology on the coefficients of the mapping vector **u**. Unlike Lasso [43] where a solution path can be found via varying a parameter controlling the L1-norm sparsity, here we impose a quadratic form penalty similar to L2-norm ridge regression [44]. Thus, the solution defines a rank order (based on **u**’s elements) of network edges. The optimal value for *t* is selected via cross-validation which is typical for such settings [21, 44]. More specifically, we tune not only *t*, but also the number of selected edges based on grid search using an inner cross-validation on the training data. The first constraint in Eq (1) is similar to ridge-shrinkage in linear regression [44] based on the graph topology, and thus its purpose is to “shrink” values of irrelevant vertices to zero, while the second constraint removes the scale freedom of vector **u** to ensure uniqueness of the solution. Detailed derivations and algorithmic solutions for optimizing this objective function can be found in [21].

Compared to recent alternative techniques [37, 38], SNL always produces a unique solution and more importantly, its solution is globally optimal with respect to the optimization function. Specifically, one alternative method, termed MINDS [37], is based on Markov Chain Monte Carlo (MCMC) while while another, called NGF [38], relies on a heuristic sampling procedure from the exponential space of all possible subgraphs. Both produce unstable results across runs due to their sampling from a large (exponential) space of possible subgraphs. These alternatives are, thus, less suitable for selecting consistent subgraph biomarkers within cohorts.

For our task of *learning rate* classification, we learn the projection vector **u** using SNL and further threshold it to obtain a subset of the features. A linear classification model based on the retained features produces on average cross-validation accuracy of 81%. In contrast, a widely adopted support vector machine (SVM) classifier presented with the full set of 6216 features/vertices achieves 76% classification accuracy. Note that both results are based on 9-fold cross validation of the session instances. The reason for this difference is that SVM does not take advantage of the inherent graph structure among features which in our case model coherence values. Importantly, SNL outperforms SVM while using only a small subset of the features (i.e., those included in the discriminative subgraphs), while SVM uses all features. While feature selection can be performed as a preliminary step to improve SVM’s accuracy, off-the-shelf feature selection methods cannot enforce graph connectivity of the selected features. On the contrary, SNL optimizes classification accuracy and connectivity of employed features simultaneously. Hence, beyond classification accuracy, we employ SNL as a feature selector in order to identify candidate subnetworks that we then subject to significance testing, retaining only conserved and statistically significant subnetwork biomarkers. SNL’s source code is available at http://www.cs.ucsb.edu/~dbl/publications.php.

### Significant biomarkers

Our substructure mining approach extracts a set of connected discriminative subgraphs based on the classes of global network state instances. For our application, one can view SNL as a feature selection method with regularization based on the graph structure captured using edge coherence values. When the number of training instances is small and the instances are high-dimensional, feature selection approaches may suffer from overfitting to the training data. Our goal, however, is to discover biomarker subgraphs whose predictive power is expected to generalize to novel unseen instances of functional networks and is also statistically significant. To achieve these desirable properties, we perform subgraph selection multiple times for random subsets of the training instances and focus on conserved subgraphs that are consistently selected. This approach is conceptually similar to Bootstrap aggregation [45] with the distinction that our goal here is to detect a stable subset of discriminative features as opposed to combine the predictions of multiple classifiers. Therefore, since there is no available ground truth for biomarkers (i.e. functional edges that are guaranteed to be associated with learning), conventional quality measures like sensitivity, specificity and ROC curves are not applicable for quantifying the quality of the outcome. Instead, we evaluate the statistical significance of the predictive power of conserved subgraphs using a *q*-value statistic that implements a strict false discovery rate correction for multiple comparisons [23].

**Conserved subgraphs.** A common machine learning approach used to improve the generality and stability of a learned model is Bootstrap aggregation [45], where multiple versions of a training set are generated and the individually trained models are aggregated to produce a single model. Our method generally follows this strategy. However, unlike Bootstrap aggregation which samples the data with replacement and actually is an ensemble method, we train our models based on cross validation and perform such cross validation multiple times in order to evaluate the consistency of the uncovered subnetworks. Specifically, we perform 9-fold cross validation 5 times (45 training sets in total) and select candidate biomarker subgraphs based on their consistency in cross validation, i.e., subgraphs conserved over multiple training runs. We also ensure connectivity in the resulting candidate biomarker subgraphs, as our goal is to capture differences in coordination among communicating brain regions involved in a common cognitive function or neurophysiological process (further details available in S1 File).

The problem of obtaining conserved subgraphs using SNL is similar to frequent connected subgraph mining (FSM) [46]. Given a database of subgraphs and a frequency threshold, the goal of FSM is to compute connected subgraphs that appear more frequently in the database than a specified frequency threshold [22]. For our setting, such subgraphs will appear in at least a pre-specified number of trained models (i.e., subgraphs obtained by SNL). The rich literature on the general problem of frequent subgraph mining includes applications to computational chemistry, program analysis, and others with multiple proposed variations of the problem and corresponding methods [46]. For our analysis, we employ gSpan [22], a commonly used and computationally efficient approach for general subgraphs. The input to the algorithm is the set of possibly disconnected subgraphs obtained over multiple runs of SNL and a frequency threshold. For our analysis, we require that subgraphs are selected in at least a third of the runs of SNL. Less conservative frequency thresholds, significantly increase the number of candidate subgraphs but do not increase the number of subgraphs that pass the significance test (described next). Further details on this step of the analysis are provided in the Supporting Information (S1 File).

**Testing the statistical significance of conserved subgraphs.** To evaluate the significance of the individual discriminative power of the obtained conserved subgraphs, we compute their *q*-values [23] with respect to a random population of connected subgraphs of matching size. We choose the *q*-value as our significance measure as it reflects the false discovery rate (FDR), as opposed to the false positive rate (FPR) captured by *p*-values. The *q*-value measure of statistical significance has been employed for genome-wide studies and has significant advantages over alternative corrections for FDR [23].

One challenge in computing the *q*-value for our subgraphs is that we need a background model of expected discriminative power of random graphs. To estimate this background model for subgraphs of varying number of edges, we sample connected graphs of fixed size uniformly at random and compute their accuracy in classifying all training instances based on an SVM classifier with polynomial kernel, involving the corresponding subset of features. To ensure uniform sampling of connected subgraphs, we employ a random-walk based sampling technique with degree-based rejection [47, 48] (details available in S1 File). To estimate the *p*-values and subsequently *q*-values for our conserved subgraph candidates, we use the corresponding background accuracy distributions. We retain subgraphs of *q*-value ≤0.015, thus the FDR for our selected subgraphs is 0.015 [23].

## Results

We apply our biomarker mining technique to the fMRI data acquired during the sensorimotor learning task described above consisting of session-specific functional networks coupled with global states: high and low learning rates. We discover 21 subgraph biomarkers that are both conserved in the sets of candidates produced by SNL and whose individual accuracy is significant (*q*-value ≤ 0.015). These candidate subgraphs are selected based on their consistent detection (at least in a third of all runs) in 9-fold cross validation performed 5 times where the optimal parameters are chosen based on the training accuracy and tested on the left-out fold according to [44]. The sizes of the subgraphs range between 1 and 5 edges which connect 2 to 6 brain regions. Their individual training accuracy ranges between 74% and 85% (based on an SVM with a polynomial kernel). The list of all conserved and significant subgraphs as well as their individual accuracy and significance is provided in the Supporting Information (S1 File). These subgraphs share edges and larger substructures, i.e., there is redundancy in the brain regions and connections they cover. We, thus, focus our analysis on the union of their edges which corresponds to two disjoint connected regions.

**Fig 3:**
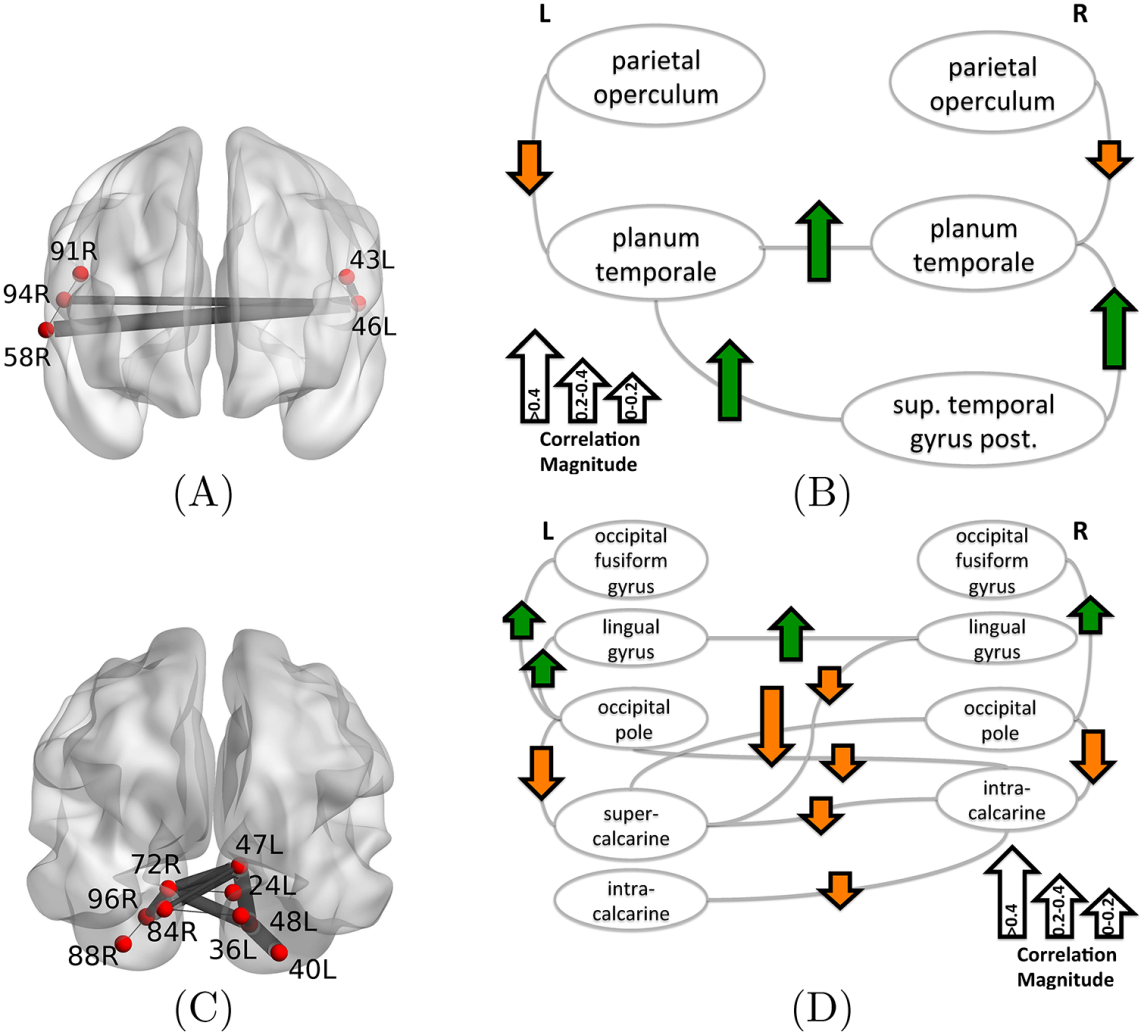
Discriminative biomarker regions related to learning formed as the union of edges in individual biomarkers. (A) The bilateral superior temporal-parietal (BSTP) biomarker region involves planum temporale, parietal operculum and the right superior temporal gyrus, while (C) the bilateral occipito-temporal (BOT) biomarker region resides predominantly in the occipital cortex, involving vision-related regions. The biomarker regions are visualized both superimposed on the brain (A), (C) and as logical graphs (B), (D). The latter also shows the correlation between edge coherence and the learning rate of task completion (green upward arrow means the edge is coherent in sessions with high learning rate). Mapping of all brain region ids to anatomical names is provided in (S1 File).

Fig 3 shows the two biomarker regions (mapping of all brain region ids to anatomical names is provided in S1 File). The first biomarker is the bilateral superior temporal-parietal (BSTP) biomarker (Fig 3A), and it involves bilateral planum temporale and parietal operculum as well as the right superior temporal gyrus (the posterior portion). We represent the correlation between edge coherence and learning rate in Fig 3B. The arrows associated with edges capture both the correlation sign (positive correlation corresponding to upward green arrow) and the magnitude (arrow length). In the case of the BSTP biomarker (Fig 3B), the dominant correlation is positive and in particular higher coherence in the circuit composed of the left and right planum temporale and the superior temporal gyrus corresponds to sessions of high learning rate.

Particularly interesting in the bilateral superior temporal-parietal biomarker is the involvement of the parietal operculum whose coherence with planum temporale has a negative correlation with learning rate. Notably, the parietal opercular cortex is involved in manipulation and macroscopic tactile sensation [25, 49], processes that are critical to participants learning this task which requires a growing familiarity with the keyboard and the mapping of stimuli to movements. Parietal opercular cortex is also involved in predicting sensory consequences of motor commands [26, 50]. In light of these roles for the parietal operculum in motor tasks, we can now interpret the BSTP biomarker. The learning rate is high when the earlier required dependence on simple sensory motor mapping has been completed, and therefore parietal operculum is no longer needed.

The second biomarker is predominantly in the occipital lobe (Fig 3C) and we will refer to it as the bilateral occipito-temporal (BOT) biomarker. It interconnects the bilateral occipital fusiform gyrus, lingual gyrus, occipital pole, and intracalcarine cortex, as well as the right supercalcarine cortex. All of the above regions are involved in visual processing. Overall, the coherence between the cortical regions in the left and right hemisphere is negatively correlated with learning rate. This means that there is communication among visual cortex regions in the early sessions when subjects are still getting familiar with the visual cues and do not register high rates of task completion. This visual cortex coordination is reduced in high learning rate sessions. An exception to that trend is the circuit involving the left and right lingual gyrus and its coherence with the occipital poles and occipital fusiform gyri. This circuit is more coherent in high learning rate sessions and less coherent in low learning rate sessions. Unlike the calcarine cortices, the lingual and fusiform gyrus are higher order visual areas. We speculate that the nature of the task—visually decoding tablature with 12 different colored notes—is highly dependent on these higher order visual processing areas. Stronger coherence among these areas would lead to faster learning.

The CSP task requires subjects to plan sequential movements from relatively complex visual stimuli. Interestingly, we found that subjects with more shallow learning curves had greater connectivity of primary visual cortical areas, typically involved in processing of low level visual features. On the other hand, we found that steeper learning curves were correlated with increased connectivity in higher-order visual regions, regions which are involved in visual object recognition [51], including words [52]. These results indicate that greater synchrony in higher-level visual regions supports quicker learning in particular, when learning a motor skill involves the parsing of complex visual stimuli. This suggests that greater connectivity of higher-level visual regions involved in recognition might signify the perception of sequential note patterns as unique motor sequence identifiers and less as collections of individual notes.

It is important to note that the available training data in our learning task do not deem any significant and predictive bridging edges/paths that connect the two biomarker regions. Under more observations or different learning tasks the two biomarker regions may merge in one or may change in terms of edge and region memberships.

All distinct biomarker edges are also listed in Table 1 together with corresponding statistics such as average coherence in low and high learning rate sessions and the actual value of correlation with learning rate. Additional analysis and discussion of the two biomarker regions follows in the subsequent sections.

**Table 1:** Summary statistics of the selected biomarker edges. The first two columns show the region names and involved functional edges within the 2 biomarker regions. The next three columns show the average coherence values in high-rate and low-rate learning sessions, respectively. The last column shows the correlation of edge coherence and the session learning rate.

Region	Functional edge	Avg-high	Avg-low	Correl. with learn. rate
BSTP	R sup. temporal gyrus, post / R planum temporale	0.531	0.466	0.523
	L planum temporale / R sup. temporal gyrus, post	0.370	0.296	0.492
	L planum temporale / R planum temporale	0.419	0.307	0.467
	L parietal operculum / L planum temporale	0.422	0.432	-0.087
	R parietal operculum / R planum temporale	0.446	0.502	-0.241
BOT	L lingual gyrus / R lingual gyrus	0.699	0.652	0.298
	R occipital fusiform gyrus / R occipital pole	0.553	0.446	0.191
	L occipital fusiform gyrus / L occipital pole	0.576	0.444	0.164
	L lingual gyrus / L occipital pole	0.462	0.401	0.005
	L supercalcarine cortex / R lingual gyrus	0.43	0.457	-0.0293
	L intracalcarine cortex / R intracalcarine cortex	0.623	0.626	-0.0749
	L supercalcarine cortex / R intracalcarine cortex	0.546	0.598	-0.152
	L occipital pole / R intracalcarine cortex	0.390	0.368	-0.187
	L supercalcarine cortex / L occipital pole	0.309	0.344	-0.264
	R intracalcarine cortex / R occipital pole	0.386	0.383	-0.299
	L supercalcarine cortex / R occipital pole	0.296	0.356	-0.419

### Differences in high- and low-rate learning sessions

**Fig 4:**
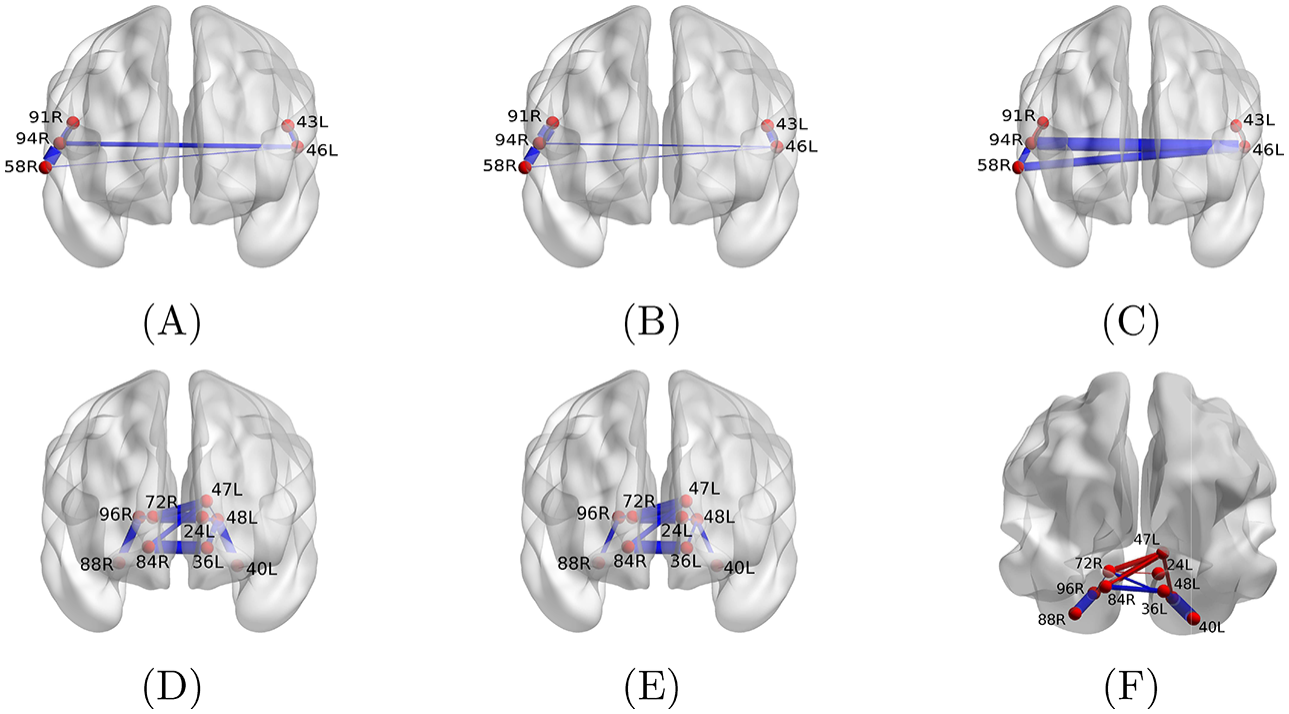
Average coherence of edges between high- and low-rate learning sessions in the two biomarker regions. Edge thickness encodes the average coherence in high (A), (D) and low (B), (E) learning rate sessions, as well as the absolute difference between the averages (C), (F). Furthermore, the blue color in (C), (F) designates the average high-rate learning coherence being larger than that of low-rate learning and the red color shows the opposite case. Mapping of all brain region ids to anatomical names is provided in S1 File.

Biomarkers are discriminative between learning rates based on their individual edge coherence values. However, to better understand the potential differences in their coordination of distributed neural circuits, we next examine the biomarkers’ average coherence profile patterns. We visualize both biomarkers in their anatomical locations in the brain, and we vary the thickness of the edges to represent the average coherence observed in high (Fig 4A and 4D) and low (Fig 4B and 4E) learning rate sessions. The actual average values and their difference are also reported in Table 1. The most salient features of high-rate learning sessions associated with substantially *high* coherence are edges 40L/48L (left occipital fusiform gyrus—left occipital pole), 88R/96R (right occipital fusiform gyrus and right occipital pole) and 46L/94R (left and right planum temporale). These areas are key players in visual processing and color recognition [27], critical cognitive processes required by the task.

The most salient features of high-rate learning sessions associated with substantially *lower* coherence are edges 47L/72R (left supercalcarine cortex and right intracalcarine cortex), 47L/96R (left supercalcarine cotex and right occipital pole) and 91R/94R (right parietal operculum and right planum temporale). In Fig 4C and 4F, we display edges with reduced coherence in high learners in red and edges with increased coherence in high learners in blue; the thickness of each edge corresponds to the magnitude of the difference.

## Discussion

Identifying the neural-circuit level drivers of higher order cognitive processes in humans is a critical frontier in human neuroscience. Reaching this goal will require concerted efforts across many fields of science, leading to the development of technologies from novel imaging techniques to new powerful computational tools that can extract both descriptive statistics and predictive features. We seek to address the latter challenge by offering a methodological approach to identify discriminative network biomarkers in the form of connected subgraphs that can be used to separate non-invasive neuroimaging measurements according to cognitive performance. Our approach capitalizes on recent advances in computer science and graph mining, and is accompanied by strict statistical testing and validation. We exercise our approach in the context of visuo-motor skill learning, and uncover two predictive biomarkers that distinguish high from low learning rate training sessions. Together, the method and application offer an important perspective on higher-order cognition in humans, and provide a generalizable toolset for use in other studies.

### Visuo-motor learning as a network process

Traditionally, visuo-motor learning in both humans and animals has been studied at a relatively local level from the general perspective of brain mapping. In this view, regions of the brain, or ensembles of neurons, are identified as physical volumes whose activity may change as the animal learns. This approach has led to the extensive insights that build our intuitions about motor learning today [30]. However, in recent years, this regionally-focused view has begun to be complemented by other perspectives highlighting the fact that changes in time series properties [53], functional connections [54], or even large-scale connectivity patterns may each play a role in neural computation and its relationship to behavior [55].

Focusing on this latter approach, previous network-based studies of visuo-motor learning in humans in the specific context of finger sequences have delineated several features of the network dynamics or reconfiguration properties associated with learning [3, 17, 56–58]. These studies together demonstrated that temporal changes in the community structure and centrality of brain regions (network nodes) can be detected in networks estimated from functional magnetic resonance imaging, complementing prior work in electrophysiological measurement modalities such as EEG and MEG [59]. Here we take a complementary approach and instead ask the question of whether we can identify local subnetwork patterns that can predict high from low learning, irrespective of the time at which that learning occurred (e.g., early *versus* late in training). We address this question by comparing and contrasting the brain network structures observed in groups characterized by low *versus* high learning rate, and thereby identifying the most discriminative subnetworks. The uncovered patterns are thus in the form of local interactions among functionally correlated brain regions, and thus provide a deeper understanding of the local network structures facilitating visuo-motor learning in healthy adult humans.

Our approach—deeply steeped in recent advances in computer science and graph mining—complements other recently developed statistical approaches. For example, Kim and colleagues recently proposed a set of statistical tests for comparing the functional connectivity between brain (or mental) states [13, 14]. The authors employ sparse matrix estimation based on the *graphical lasso* [60] by imposing a fixed level of sparsity within a set of observations of one kind (e.g., disease or control), followed by regression and normalization of individual edges [13]. Using this preprocessed data, the authors then apply the spatial pairwise clustering approach [12], or the network based statistic approach [11] to test for network differences between classes. Importnatly, this analysis of Kim et al. enables a robust characterization of individual essential edges. Here, by comparison, we focus on the discovery of connected subgraphs that are discriminative using edges scores of both classes simultaneously. Our framework is, in a sense, orthogonal to the statistical tests for individual edges, in that the scores estimated by Kim et al.’s methodology can serve as features in our methods instead of raw coherence values. The novelty of our approach comes from enforcing connectivity of subgraphs (as opposed to disjoint edges) in both the discovery process and in the subsequent significance tests. That is, we test subgraph significance as opposed to individual-edge or all-edges significance.

Our study also differs from prior work building on this same data, which has predominantly focused on extracting network patterns that distinguish good from poor learners in a purely descriptive manner that did not incorporate any sophisticated tools from machine learning [57, 58]. Indeed, the aforementioned studies focused on describing global network changes as a new skill was acquired over an extended training period of 6 weeks. For example, the results described in [57] reveal that the core-periphery organization [61] of the brain can predict individual differences in extended learning estimated from out-of-scanner behavior. More specifically, good learners were more likely to have a greater separation between the network core and the network periphery than poor learners. In a more recent study [58], the group sought to identify the fundamental functional modules present during visuo-motor skill learning, and reported a growing autonomy of these systems as participants acquired the new skill. In contrast to these descriptive approaches, the subgraph biomarker analysis that we present here offers an alternative lens in which to understand the differences in learners in a long-term training setting. Indeed, rather than identifying meso-scale structures such as communities, or macro-scale features such as centrality, this approach identifies sparse, local network motifs or subgraphs whose pattern of coherence can predict the behavioral outcome. In other words, this approach offers a much more parsimonious account of network characteristics supporting human learning.

### Biomarker regions, learning and further interpretation

The human parietal operculum (OP) is a heterogeneous cortical area overlapping with multiple Broadmann regions [62, 63]. Evidence shows that it contains at least 2 sensory representation maps of the body and is involved in the processing of somatosensory information, responding to both non-noxious and noxious stimulation [64, 65]. Moreover, this region is involved in proprioceptive feedback during active movements, with recent work suggesting that this region is involved in the coordination of finger movements [66, 67]. The planum temporale (PT) is considered secondary auditory cortex and is functionally involved in higher order auditory and language processing, including speech, reading, and auditory-motor integration [68]. Together, converging evidence suggests that this region acts as a specialized hub for spectrotemporal processing of stimuli [69]. We found that reduced connectivity between the PT and OP was related to faster learning. This suggests that a greater independence of specialized hubs, one that is involved in hand related sensorimotor feedback (OP) and the other, in spectrotemporal processing (PT), served to promote learning. Moreover, greater cross-hemispheric connectivity of the PT with other neighbors, might serve to strengthen learning as well. In this regard, we presented participants with stimuli that represented a music-like notion, which required participants to “read” the notes, and map vertical and horizontal position to the appropriate finger. So, it is possible that greater connectivity with PT leads to swift learning, which could reflect a greater proficiency in translation of the music staff to motor output. It is unlikely that this effect is due to individual differences in music training because we selected participants with minimal music experience (less than 4 years total). More generally, the relationship between individual differences of learning rate and local functional connectivity is consistent with an emerging literature that considers the evolution of brain activity across networks rather than local regions. Historically, both increases and decreases of regional activity have correlated with amount of training, but not individual differences of learning rate [70]. With the development of functional connectivity metrics, it was evident that local, pairwise connectivity could also change with learning [20, 71] and correlate with depth of knowledge. In recent studies that consider the evolution of functional connectivity across larger brain networks, it has become apparent that the strength of connectivity, both positive and negative, between separable network communities can evolve with training [31]. Allegiance of a network node to a community, as well as allegiance between networks are strong predictors of learning as well as individual differences in the rate of learning [58]. Indeed, it has been possible to show that over-involvement of prefrontal areas associated with executive control are associated with slower rates of learning, presumably by delaying the emergence of autonomous motor behavior. In this context, the current results provide additional complementary evidence by demonstrating that increased connectivity between secondary somatosensory cortex and the temporal cortex associated with abstract sequential information can also result in slower learning. Whether this is due to a delay in the development of autonomous motor behavior, a competition between brain systems that represent sequential information differently or some other process remains to be determined.

### Methodological considerations

There are several important methodological considerations pertinent to this work. First, it is important to note that the methodological approach that we develop and apply in this work—based on subgraph biomarker mining—does not consider dynamic or time-evolving aspects of the functional interactions. However, extensions of the approach that we develop here to time-evolving networks could be particularly useful in studying the ability of a brain region to broadcast or receive information in learning tasks. Such extensions are likely possible by building on the recent methods for extracting significant dynamic subgraphs from temporal networks of various genres [72, 73]. Indeed in future, it will be particularly interesting to ask how to mine neuroimaging data for significant subgraph biomarkers as the functional (or structural) networks evolve in time. Answering this question could offer an important view into the dynamics of circuit function essential for human learning.

A second important consideration lies in the empirical challenges inherent in collecting long-term training data. In this study, we used data acquired in 3 sessions spaced over 5 days from 18 healthy adult individuals. Such a longitudinal study is extremely difficult to complete in terms of recruiting, cost, and personnel resources, and is therefore a particularly valuable resource. Nevertheless, it would be important in future to validate our results on similar longitudinal data sets acquired in a separate set of healthy adult human subjects.

A third important factor that deserves consideration is the length of training time. We kept each practice session below 1.5*hr* to decrease potential for fatigue, thus extending our study over 3 days to adequately sample of the first, fast rate of improvement characteristic of early motor skill learning. We anticipate that the extracted biomarker is relevant for the initial stages of learning, but we cannot claim that this same biomarker would be identified if we studied longer term learning, where other cognitive processes are thought to be involved [30].

Another consideration of interest is that the discriminative subgraphs that we identify in this work are likely to be highly-specific to the particular visuo-motor task performed. Other types of learning tasks, and even other types of visuo-motor tasks (such as visuo-motor tracking) may display different discriminative subgraphs. It will be particularly interesting in future to catalogue the discriminative subgraphs that distinguish high *versus* low cognitive performance or task performance across a range of motor and non-motor learning tasks.

### Limitations

There are several limitations of the current imaging and analysis, that will be important to address in the future via additional experimentation and generalized computational analysis. One important limitation of the neuroimaging component is that the acquisition was performed without multiband technology, thus decreasing the temporal resolution of the data. It would be useful in future studies to use recently developed higher-resolution temporal sampling techniques [74] to increase the statistical power to detect individual differences in neural markers of learning.

The complexity of the experimental task resulting in a limited number of subjects and respectively learning sessions is another limitation. While we have taken extensive measures to alleviate this drawback: (i) repeated cross validation with fold re-sampling, (ii) L2 regularization to increase the stability of selected subgraphs and (iii) subsequent subgraph statistical significance testing based on q-values, we expect that larger datasets will enable even more stable and statistically significant biomarkers. Beyond more data, further computational extensions can be considered to further alleviate the relatively small number of instances compared to features. In future studies, we will seek to incorporate L1-norm constraints which can shrink coefficients of irrelevant edges to zero (sparsity).

Another improvement may be enabled by adopting structural connectivity maps as priors for coordination among regions as opposed to solely the observed functional coherence from fMRI [75]. Such analysis will benefit from low level of spurious interactions due to noise, imaging artifacts or concurrent processes in the brain, but will require diffusion imaging scans for the participating subjects.

## Conclusions

We developed a general approach for the discovery of brain subgraph biomarkers from fMRI data associated with global labels. Our approach is based on discriminative subspace learning in network space coupled with significant conserved subgraph mining. We applied our method to data acquired during the performance of a sensorimotor learning task. We obtained two significant biomarkers involving circuits related to visual processing, motor performance, and learning, which together suggest novel interactions among regions that may play a critical role in visuo-motor skill learning. While we focused on data from a learning experiment as a case study for our method, our framework can be applied to a variety of settings. Beyond analysis of other cognitive tasks, one can also adopt our method to detect biomarkers specific to neurological and psychiatric diseases, by applying the method to fMRI data acquired in patients and controls.

## Supporting information

S1 File Supporting information file. (PDF)
