# Supplementary material for: Learning about learning: Mining human brain sub-network biomarkers from fMRI data
Source: PLoS One. 2017 Oct 10;12(10):e0184344. doi: 10.1371/journal.pone.0184344 (PMC5634545; doi:10.1371/journal.pone.0184344)
Supplement: S1 File — (PDF) [file pone.0184344.s001.pdf]

Learning about Learning: Mining Human Brain Sub-Network Biomarkers from fMRI Data (Supporting Information)

Global Learning Rate States

The network state mining methodology we adopt works with categorical labels of the global states, i.e. it is formalized as an instance of classification as opposed to one of regression. While estimating continuous global scores (regression) might be of interest as well, here we focus on identifying biomarkers that differentiate between sessions in which subjects progressively increase the speed of completion of the visual cues (high learning rate sessions) from those in which no acceleration is observed (low learning rate sessions). We adopt the slope of the movement time over trials practiced as a measure of the learning rate. Movement time is the time between the first and last button press of the 12-note sequence. A larger slope indicates that the subject is learning well, while a smaller slope indicates that the subject is not learning as well [1].

The slopes for the 18 subjects are listed in Table 1. The coloring of scores in the table are based on a threshold of -0.06 (which we discuss in more detail in the next paragraph) used to convert the continuous slopes into the categorical low versus high learning rate states. Slopes smaller than the chosen threshold correspond to high learning rate (blue) and slopes larger than the threshold correspond to low learning rate (yellow). In the first session, 13 of the subjects are learning the motor task as their rates are below the threshold, while 5 of the subjects are not learning as fast. We focus on the fast early changes in the learning process as posited in the theoretical framework of Doyon et al. [2]. Thus the 13 subjects that were labeled as being in a high learning state in the first session were discarded from the second and third session data sets. In the third session, 1 subject transitions from a low to a high learning rate state while the remaining 4 low learning rate subjects remain in the low learning rate state. The confidence intervals of the slope included 0 for subject 16 in Session 2 and subject 1 in Session 3; hence, these sessions were excluded as no significant learning occurred. Note that our estimate of learning rate is independent of how fast the the subject performs at the beginning of the session, a feature which is often largely driven by biomechanics rather than the ability to learn. Instead, we focus on the *improvement* of individual performance over time, which is attributed to early-stage motor skill learning [2].

Table 1. Slopes of movement time versus trials practiced for each subject and each experimental session. High-learning rate sessions are colored blue and low learning rate sessions are colored yellow. All but five subjects exhibit a high learning rate in the first session. One of those five subjects transitions into a high learning rate in their third session. Slopes for two of the sessions (Subject 1 Session 3 and Subject 16 Session 2) were negative and thus indicated worsening of performance (colored gray).

	1	2	3	4	5	6	7	8	9	10	11	12	13	14	15	16	17	18
**Session 1**	-.12	-.10	-.05	-.09	-.09	-.07	-.04	-.13	-.07	-.07	-.07	-.13	-.04	-.08	-.10	0	-.04	-.11
**Session 2**	-.01	-.03	-.01	-.03	-.06	-.04	-.03	-.05	-.02	-.04	-.05	-.03	-.04	-.04	-.02	NA	-.04	-.03
**Session 3**	NA	-.03	0	-.1	-.07	-.05	-.07	-.08	-.02	-.01	-.02	-.03	-.04	-.03	-.01	-.05	-.03	-.03

We selected a threshold of -0.06 on the learning rate to delineate high versus low learning rate states. To choose this threshold, we used a clustering approach with perturbations and we estimated the optimal threshold between states based on the

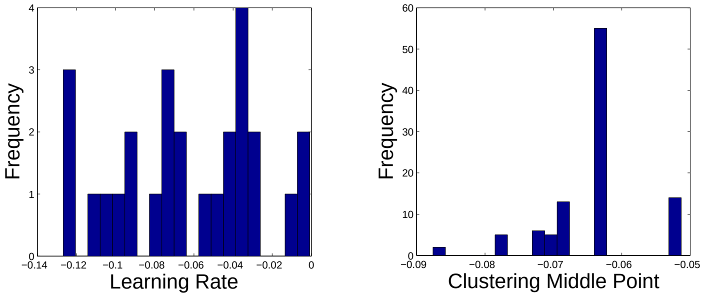

(a) Stability of top regions (b) Stability of top edges

Fig 1. Distribution of the learning rate in sessions of interest (a) and middle clustering point distribution (b) when clustering the values 100 times applying a perturbation noise of 3 standard deviations of the original values.

stability of multiple clustering solutions. To begin, we randomly perturbed the measured learning rates from all valid sessions by adding Gaussian noise with a magnitude of 3 standard deviations of the original learning rates to each parameter value. We created 100 perturbed instances of the measured learning rates and clustered them using k-means clustering (at $k = 2$). We compared the different clustering solutions using the Jaccard index as a measure of similarity. A clustering is considered "stable" if it is approximately maintained across multiple perturbations. We observed that the vast majority of clustering pairs had a Jaccard index of 1 (perfect similarity) and -0.06 is the middle point separating the points in the obtained clusterings. We show the distribution of the original learning rate values in Fig. 1(a) and the distribution of the middle point between two perturbed clusters in Fig. 1(b). Both figures single out -0.06 as an optimal threshold.

Using the selected threshold and discarding sessions preceded by high learning rate sessions of the same subject, we obtained 14 high learning rate session-specific networks and 13 low learning rate session-specific networks (color-coded with blue and yellow respectively in Tab. 1). We applied our discriminative biomarker approach considering all 27 networks simultaneously together with their global learning state labels.

Multiple thresholds, and thus, multiple classes corresponding to levels of learning rate, can be selected similarly using clustering. Such modeling of more than two classes is possible within our SNL learning framework as we discussed earlier. Note that the two meta-networks described earlier, will still be suitable in this case since their topology is determined by network samples having the same or different global state values and not on their specific class labels. The number of dimensions for the subspace in which SNL projects the original data will also have to be adjusted to be one less than the number of classes. Moreover, as there will be a natural order of the classes (e.g., gradients of learning rate), the penalty for rendering different-class instance pairs close should be scaled according to the distance between their classes in that natural order. The limited number of subjects and training sessions in our data, however, drove the decision to work with two classes for this specific evaluation task.

Discriminative subspace mining: parameters and comparative analysis

As a baseline, we compared the testing accuracy of SNL with that of an SVM classifier that works with the same instances, but which is oblivious to the network structure. All 6216 features were given to the SVM while performing cross validation. We also used the linear-SVM as the classifier for SNL but it performed on the subspace spanned by the subnetworks selected by the SNL. The original data set of 27 samples was randomly divided into 9 folds and in the cross validation (CV) model, we used each of the 8 folds as a training data set while the left-out fold was used as an independent test set. This 9-fold cross-validation was further performed 5 times with different random data splitting and we report the averaged prediction accuracy from 45 testing folds. While the conventional SVM technique achieved 76% testing accuracy using all features, SNL achieved 81% using only the top 50 selected features.

Conserved connected discriminative subgraphs

The result of SNL's discriminative subspace learning was a set of 45 edge subsets, one from each training version. Each of those selected edge subsets did not necessarily form a single connected component. There were a total of 456 connected components in the 45 selected edges sets. The maximum number of occurrences for any subgraph of the selected edges was 45 and hence when we mined the space of subgraphs for conserved ones, we needed to define a support threshold, i.e. a number of occurrences out of the 45 possible.

Table 2. Number of candidate frequent subgraphs for decreasing frequency threshold.

**Frequency**	**#Candidates**
25	152
20	1387
15	7844
10	100566

In this analysis, we began with a very high threshold and lowered it to obtain a larger number of candidate biomarkers to be further tested for significance. Table 2 lists the number of frequent subgraphs in the set produced by SNL for decreasing frequency. For conservative (high) thresholds, we obtained a small number of candidates but importantly, we could easily miss candidates of lower frequency, whose accuracy may not have been significant. As we lowered the threshold, the number of candidates increased drastically, since the space of subgraphs increased exponentially. In this process of varying thresholds, we observed that the set of subgraphs with significant predictive accuracy (q-value ≤ 0.015) initially grew and for thresholds lower than 15 occurrences out of 45, we do not obtain new significant subgraphs. The reason for this phenomenon is that at lower thresholds we had many subgraphs of high overlap that satisfied the threshold, but whose individual accuracy was not significant.

Background models and subgraph statistical significance

Given a subgraph characterised by a subset of functional edges in the brain network, we quantified its discriminative power by the accuracy of classifying all training

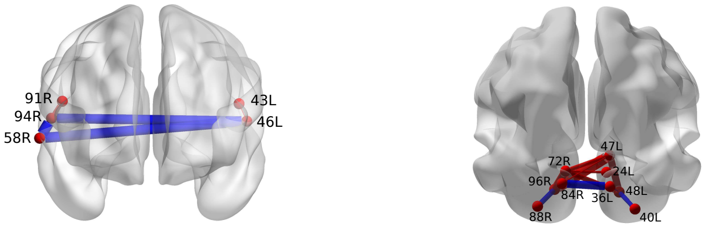

(a) Bilateral Superior Temporal-Parietal (BSTP)

(b) Bilateral Occipito-Temporal (BOT)

Fig 2. Correlation between biomarker edge coherence and the learning rate. Edge thickness encodes the magnitude of correlation while blue and red colors respectively show the positive and negative correlations.

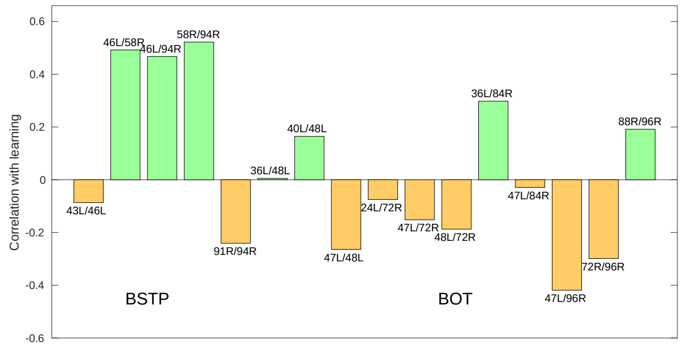

Fig 3. Correlation between each of selected edges' coherence and the learning rate. Green and orange encode positive and negative correlations respectively.

instances using only the subset of features corresponding to included edges. We used an SVM classifier with polynomial kernel for this analysis. Our goal was to select only connected subgraphs, whose accuracy was significant. Hence, we needed a background model for the accuracy levels we could expect at random for connected subgraphs. Since a closed form solution to this problem is not available, we resorted to sampling-based estimation of the background model. Note that allowing more features naturally results in higher accuracy, hence we defined background models for subgraphs of specific sizes. We sampled 1000 subgraphs of a fixed size k using a random walk based sampling that ensured that every connected subgraph had an equal chance of being included in our sample by following the techniques in [3]. We then computed the accuracy for every subgraph in the sample by building SVM classifiers. We quantified the p-value of our conserved subgraph's accuracies (obtained by frequent subgraph mining) in the background models corresponding to their individual sizes. To correct for false discovery rate (FDR), we then computed q-values based on the obtained p-values, following the protocol from [4]. The final set of significant subgraphs with q-value ≤ 0.015 is described in the following section.

Correlation of biomarker edge coherence and learning rate

In this section we provide additional visualization of the correlation of biomarker edges strengths with learning rate Fig. 2. Thickness of individual edges is proportional to the absolute value of the correlation with learning rate, while colors denote the correlation sign: red for negative and blue for positive. The same information is also quantitatively visualized in Fig. 3.

Full set of discovered biomarkers

The full set of biomarkers falling in the two biomarker regions discussed in the experimental section are presented in Table 3. Their individual accuracy (ACC) varies between 74% and 85% and it is significant: q-value ≤ 0.015. Most biomarkers have no loops, only the last two feature loops involving the right and left occipital poles, and left intracalcarine and supercalcarine cortices. The sizes of the biomarkers in terms of number of edges $|E|$ and number of nodes $|V|$ vary between 1 and 5 and between 1 and 6 respectively.

Table 3. Significant Biomarkers.

**Subgraph**	**ACC**	$\|E\|$	$\|V\|$	**FDR**
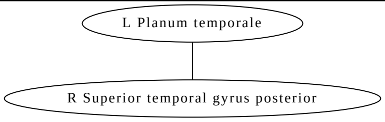	0.78	1	2	0.000
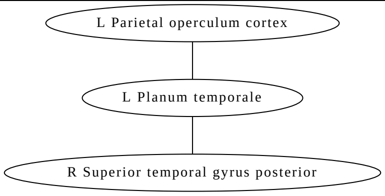	0.74	2	3	0.009
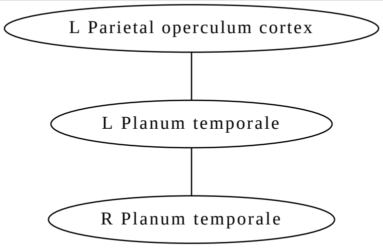	0.74	2	3	0.009
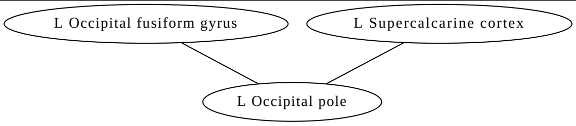	0.78	2	3	0.007
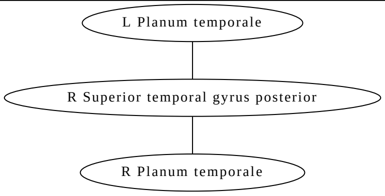	0.74	2	3	0.009

Table 3. Significant Biomarkers.

**Subgraph**	**ACC**	$\|E\|$	$\|V\|$	**FDR**
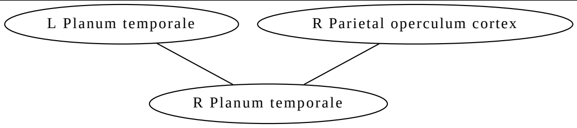	0.78	2	3	0.007
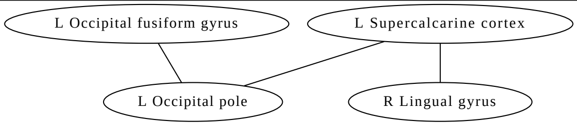	0.81	3	4	0.013
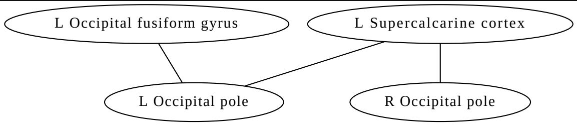	0.81	3	4	0.013
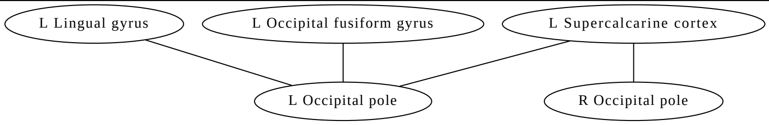	0.81	4	5	0.006
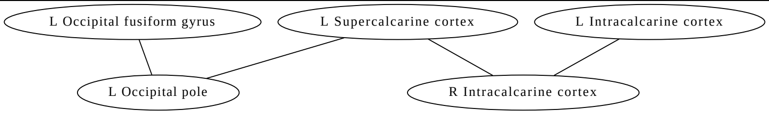	0.81	4	5	0.006
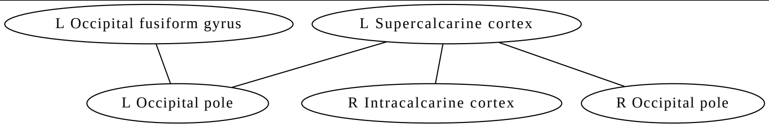	0.81	4	5	0.006
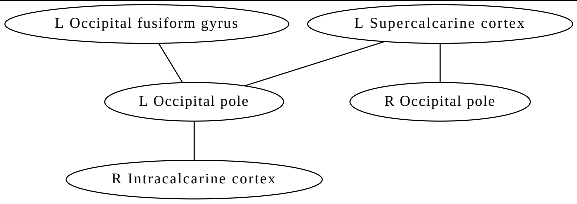	0.85	4	5	0.000
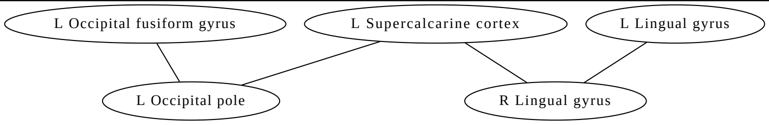	0.81	4	5	0.006
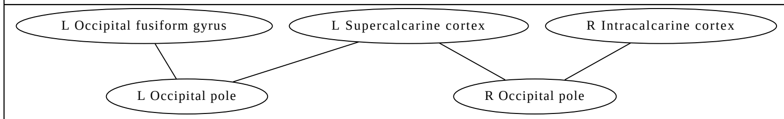	0.81	4	5	0.006
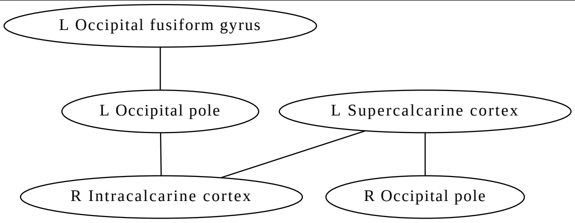	0.89	4	5	0.000

Table 3. Significant Biomarkers.

Subgraph	ACC	$\|E\|$	$\|V\|$	FDR
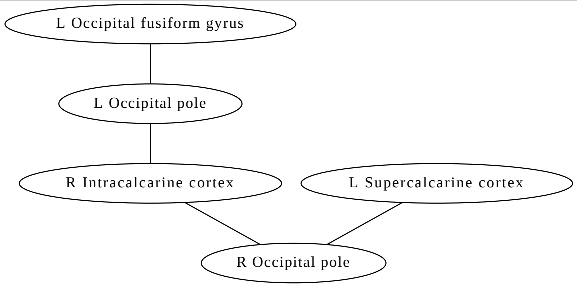	0.89	4	5	0.000
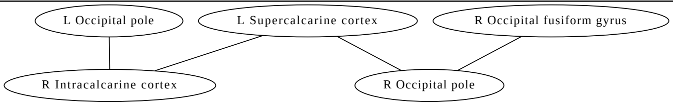	0.81	4	5	0.006
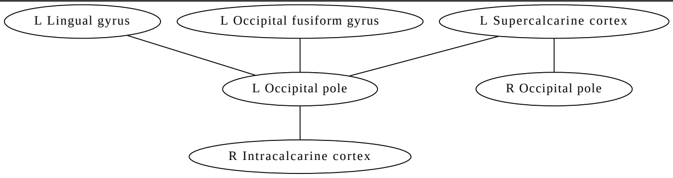	0.85	5	6	0.006
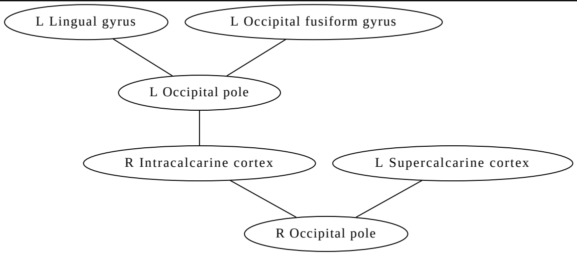	0.85	5	6	0.006
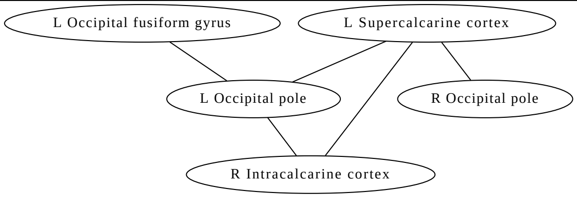	0.85	5	5	0.006
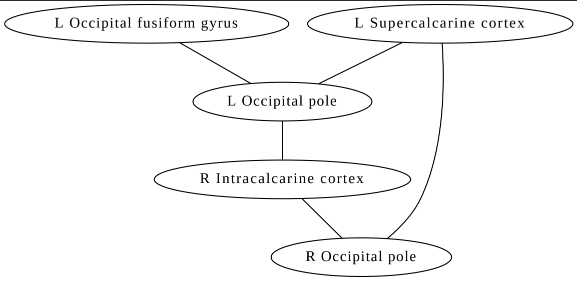	0.85	5	5	0.006

List of brain region name abbreviations

The list of all brain regions and their abbreviations used in figures throughout the manuscript is presented in Table 4.

Table 4. Brain region abbreviations.

Abbreviation	Full Name
1L	Frontal pole

Table 4. Brain region abbreviations.

Abbreviation	Full Name
2L	Insular cortex
3L	Superior frontal gyrus
4L	Middle frontal gyrus
5L	Inferior frontal gyrus, pars triangularis
6L	Inferior frontal gyrus, pars opercularis
7L	Precentral gyrus
8L	Temporal pole
9L	Superior temporal gyrus, anterior
10L	Superior temporal gyrus, posterior
11L	Middle temporal gyrus, anterior
12L	Middle temporal gyrus, posterior
13L	Middle temporal gyrus, temporooccipital
14L	Inferior temporal gyrus, anterior
15L	Inferior temporal gyrus, posterior
16L	Inferior temporal gyrus, temporooccipital
17L	Postcentral gyrus
18L	Superior parietal lobule
19L	Supramarginal gyrus, anterior
20L	Supramarginal gyrus, posterior
21L	Angular gyrus
22L	Lateral occipital cortex, superior
23L	Lateral occipital cortex, inferior
24L	Intracalcarine cortex
25L	Frontal medial cortex
26L	Supplemental motor area
27L	Subcallosal cortex
28L	Paracingulate gyrus
29L	Cingulate gyrus, anterior
30L	Cingulate gyrus, posterior
31L	Precuneus cortex
32L	Cuneus cortex
33L	Orbital frontal cortex
34L	Parahippocampal gyrus, anterior
35L	Parahippocampal gyrus, posterior
36L	Lingual gyrus
37L	Temporal fusiform cortex, anterior
38L	Temporal fusiform cortex, posterior
39L	Temporal occipital fusiform cortex
40L	Occipital fusiform gyrus
41L	Frontal operculum cortex
42L	Central opercular cortex
43L	Parietal operculum cortex
44L	Planum polare
45L	Heschl's gyrus
46L	Planum temporale
47L	Supercalcarine cortex

Table 4. Brain region abbreviations.

Abbreviation	Full Name
48L	Occipital pole
49R	Frontal pole
50R	Insular cortex
51R	Superior frontal gyrus
52R	Middle frontal gyrus
53R	Inferior frontal gyrus, pars triangularis
54R	Inferior frontal gyrus, pars opercularis
55R	Precentral gyrus
56R	Temporal pole
57R	Superior temporal gyrus, anterior
58R	Superior temporal gyrus, posterior
59R	Middle temporal gyrus, anterior
60R	Middle temporal gyrus, posterior
61R	Middle temporal gyrus, temporooccipital
62R	Inferior temporal gyrus, anterior
63R	Inferior temporal gyrus, posterior
64R	Inferior temporal gyrus, temporooccipital
65R	Postcentral gyrus
66R	Superior parietal lobule
67R	Supramarginal gyrus, anterior
68R	Supramarginal gyrus, posterior
69R	Angular gyrus
70R	Lateral occipital cortex, superior
71R	Lateral occipital cortex, inferior
72R	Intracalcarine cortex
73R	Frontal medial cortex
74R	Supplemental motor area
75R	Subcallosal cortex
76R	Paracingulate gyrus
77R	Cingulate gyrus, anterior
78R	Cingulate gyrus, posterior
79R	Precuneus cortex
80R	Cuneus cortex
81R	Orbital frontal cortex
82R	Parahippocampal gyrus, anterior
83R	Parahippocampal gyrus, posterior
84R	Lingual gyrus
85R	Temporal fusiform cortex, anterior
86R	Temporal fusiform cortex, posterior
87R	Temporal occipital fusiform cortex
88R	Occipital fusiform gyrus
89R	Fronal operculum cortex
90R	Central opercular cortex
91R	Parietal operculum cortex
92R	Planum polare
93R	Heschl's gyrus

Table 4. Brain region abbreviations.

Abbreviation	Full Name
94R	Planum temporale
95R	Supercalcarine cortex
96R	Occipital pole
97L	Caudate
98L	Putamen
99L	Globus pallidus
100L	Thalamus
101L	Nucleus Accumbens
102L	Parahippocampal gyrus (superior to ROIs 34,35)
103L	Hippocampus
104L	Brainstem
105R	Caudate
106R	Putamen
107R	Globus pallidus
108R	Thalamus
109R	Nucleus Accumbens
110R	Parahippocampal gyrus (superior to ROIs 34,35)
111R	Hippocampus
112R	Brainstem

References

1. Newell KM, Mayer-Kress G, Hong SL, Liu YT. Adaptation and learning: Characteristic time scales of performance dynamics. Human movement science. 2009;28(6):655–687.

2. Doyon J, Benali H. Reorganization and plasticity in the adult brain during learning of motor skills. Current opinion in neurobiology. 2005;15(2):161–167.

3. Li RH, Yu JX, Qin L, Mao R, Jin T. On Random Walk Based Graph Sampling. In: Proc. of International conference on data engineering ICDE; 2015.

4. Storey JD, Tibshirani R. Statistical significance for genomewide studies. Proceedings of the National Academy of Sciences. 2003;100(16):9440–9445. doi:10.1073/pnas.1530509100.
